# 1 Hz Low‐Frequency Repetitive Transcranial Magnetic Stimulation Ameliorates Epilepsy by Suppressing Interferon‐γ Signaling‐Dependent Microglial Synaptic Phagocytosis in Mice

**DOI:** 10.1002/cns.70979

**Published:** 2026-06-13

**Authors:** Donghui Lin, Duan Wang, Nong Xiao

**Affiliations:** ^1^ Department of Rehabilitation Children's Hospital of Chongqing Medical University, National Clinical Research Center for Children and Adolescents' Health and Diseases, Ministry of Education Key Laboratory of Child Development and Disorders Chongqing Key Laboratory of Child Neurodevelopment and Cognitive Disorders Chongqing China; ^2^ Zhangzhou Affiliated Hospital of Fujian Medical University Zhangzhou, Fujian province China

**Keywords:** epilepsy, microglia, neuroinflammation, repetitive transcranial magnetic stimulation, Type II interferon

## Abstract

**Aims:**

We aim to investigate the impact of low‐frequency repetitive transcranial magnetic stimulation (LF‐rTMS) on activated microglia in temporal lobe epilepsy (TLE), which exacerbate seizures and memory problems, and to explore the molecular mechanisms and clinical potential of LF‐rTMS for TLE treatment.

**Methods:**

We tested 0.3, 0.5, 1 Hz LF‐rTMS in kainic acid (KA)‐induced TLE mice, assessing EEG, inflammation, synaptic phagocytosis, memory, scRNA‐seq, and IFN‐γ neutralizing antibodies. SnRNA‐seq of human hippocampal sclerosis (HS) tissues confirmed relevance.

**Results:**

In KA‐induced TLE mice, 1 Hz LF‐rTMS decreased seizures, neuroinflammation, and hippocampal synaptic phagocytosis, while enhancing memory. These effects were linked to increased microglial IFN‐γ signaling (activating STAT2 and inhibiting CADM1/2/3), which were negated by IFN‐γ blockade. SnRNA‐seq of human HS tissues revealed elevated IFN‐γ signaling in DAM‐like cells, indicating clinical relevance.

**Conclusion:**

Our study indicates that 1 Hz LF‐rTMS is a promising treatment for TLE, potentially reducing seizures and cognitive issues by affecting microglial function. Our findings show that IFN‐γ signaling triggers STAT2 activation, which is strongly linked to the increased expression of IFN‐γ signature DAM and the decreased expression of CADM1/2/3 in DAM, thus playing a role in the neuroprotective effects of 1 Hz LF‐rTMS. These findings reveal a new LF‐rTMS mechanism and offer insights for targeted therapies in drug‐resistant epilepsy.

## Graphical abstract

In a mouse model of recurrent seizures, 1 Hz rTMS exerted neuroprotective effects by enhancing IFN‐γ‐dependent STAT2 activation in microglia and reducing CADM1/2/3 expression, thereby limiting excessive synaptic phagocytosis. Notably, co‐treatment with a neutralizing antibody against IFN‐γ abolished the 1 Hz rTMS‐induced STAT2 activation and the associated downregulation of CADM1/2/3, indicating that IFN‐γ signaling is required for the therapeutic effects of LF‐rTMS.

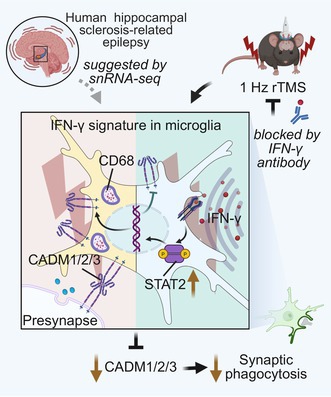

**Abbreviations**
ACSF: artificial cerebrospinal fluid
AED: antiepileptic drug
CA1: cornu ammonis 1
CADM: cell adhesion molecule
CADM1/2/3: cell adhesion molecule 1/2/3
Ccl2: C‐C motif chemokine ligand 2
Ccl3: C‐C motif chemokine ligand 3
CD68: cluster of differentiation 68
CTPA: cell type prioritization analysis
DAM: disease‐associated microglia
DEG: differentially expressed gene
DRE: drug‐resistant epilepsy
EEG: electroencephalographic
GABA: Gama‐aminobutyric acid
GFAP: glial fibrillary acidic protein
GO: gene ontology
HS: hippocampal sclerosis
IBA1: ionized calcium binding adapter molecule 1
IFN‐γ/IFN‐ II: Type II interferon
IHKA: intrahippocampal KA injection
Il‐1β: interleukin‐1β
IP: intraperitoneal injection
KA: kainic acid
KEGG: kyoto encyclopedia of genes and genomes
LF‐rTMS: low‐frequency repetitive transcranial magnetic stimulation
MDM: monocyte‐derived macrophage
MG: microglia
MGnD: neurodegenerative microglia
PBS: phosphate buffer saline
qRT‐PCR: quantitative real‐time polymerase chain reaction
RT: room temperature
scRNA‐seq: single‐cell RNA sequencing
SE: spontaneous epilepsy
SEM: standard errors of means
SMCs: smooth muscle cells
snRNA‐seq: single‐nucleus RNA sequencing
SYN1/2: synapsin1/2
TLE: temporal lobe epilepsy
Tnf‐α: tumor necrosis factor‐α
tSNE: t‐distributed stochastic neighbor embedding
UMAP: uniform manifold approximation and projection for dimension reduction

## 1 Introduction

Epilepsy affects over 70 million people globally, with around one‐third developing drug‐resistant epilepsy (DRE) that does not respond to any antiseizure drugs [1, 2]. Temporal lobe epilepsy (TLE) is a notably resistant form, where drugs mainly manage symptoms without stopping disease progression. Hippocampal sclerosis (HS), a crucial damaged area in TLE, greatly influences epilepsy progression and surgical results [3, 4, 5]. Thus, there is an urgent need to investigate alternative mechanisms of HS caused by TLE and to find effective treatments.

Recent research indicates that hippocampal local inflammatory reactions might cause damage to neurons and trigger seizures, with activated microglia potentially being key players in this process [6, 7, 8, 9]. Activated microglia disrupt the balance between excitatory and inhibitory synaptic transmission via inflammation‐mediated synaptic phagocytosis, thereby enhancing the epileptiform excitability of neural networks [8, 10, 11].

Transcranial magnetic stimulation (TMS) is a noninvasive technique that uses magnetic fields to stimulate neurons [12, 13]. Recent studies suggest that low‐frequency repetitive TMS (LF‐rTMS) may have anti‐inflammatory effects in the hippocampus by affecting microglia [14, 15, 16] and reducing excitability linked to epilepsy [17, 18, 19, 20]. LF‐rTMS alters ion distribution, influences membrane permeability, and adjusts GABA levels by increasing GAD65 expression [21, 22]. It has shown safety and therapeutic efficacy, with evidence supporting its potential in treating epilepsy [23]. After halting LF‐rTMS, seizure frequency notably dropped in chronic epileptic rats [24]. Thus, studying its anti‐inflammatory and synaptic phagocytosis effects on hippocampal microglia is crucial for understanding its therapeutic mechanisms and enhancing its use in TLE treatment.

Given the importance of stimulation frequency in rTMS effectiveness, parameter selection should be evidence‐based. Research indicates that low‐frequency rTMS, especially 0.5 and 1 Hz, consistently shows anticonvulsant effects in epilepsy models, with clinical studies supporting its therapeutic potential [25, 26]. Notably, 0.3 Hz has shown significant benefits in reducing seizures and improving cognitive and synaptic outcomes in a rat model of TLE [19, 25]. Thus, we chose 0.3, 0.5, and 1 Hz to assess their impact on pathological and behavioral outcomes in a kainic acid (KA)‐induced epilepsy mouse model. Our findings revealed that 1 Hz LF‐rTMS reduces epilepsy, neuroinflammation, microglia‐mediated synaptic phagocytosis, and memory issues. We explored the role of disease‐associated microglia (DAM) [27] in synaptic phagocytosis by enhancing IFN‐γ signaling in mouse scRNA‐seq data. Blocking IFN‐γ signaling may negate the positive effects of 1 Hz LF‐TMS via microglia. Similar IFN‐γ response changes in DAM were observed in snRNA‐seq data from patients with HS, supporting the relevance of these findings.

## 2 Results

### 2.1 1 Hz LF‐rTMS Treatment Attenuates Epileptic Seizures and Neuroinflammation in the Hippocampus of a Mouse Model of TLE

**FIGURE 1:**
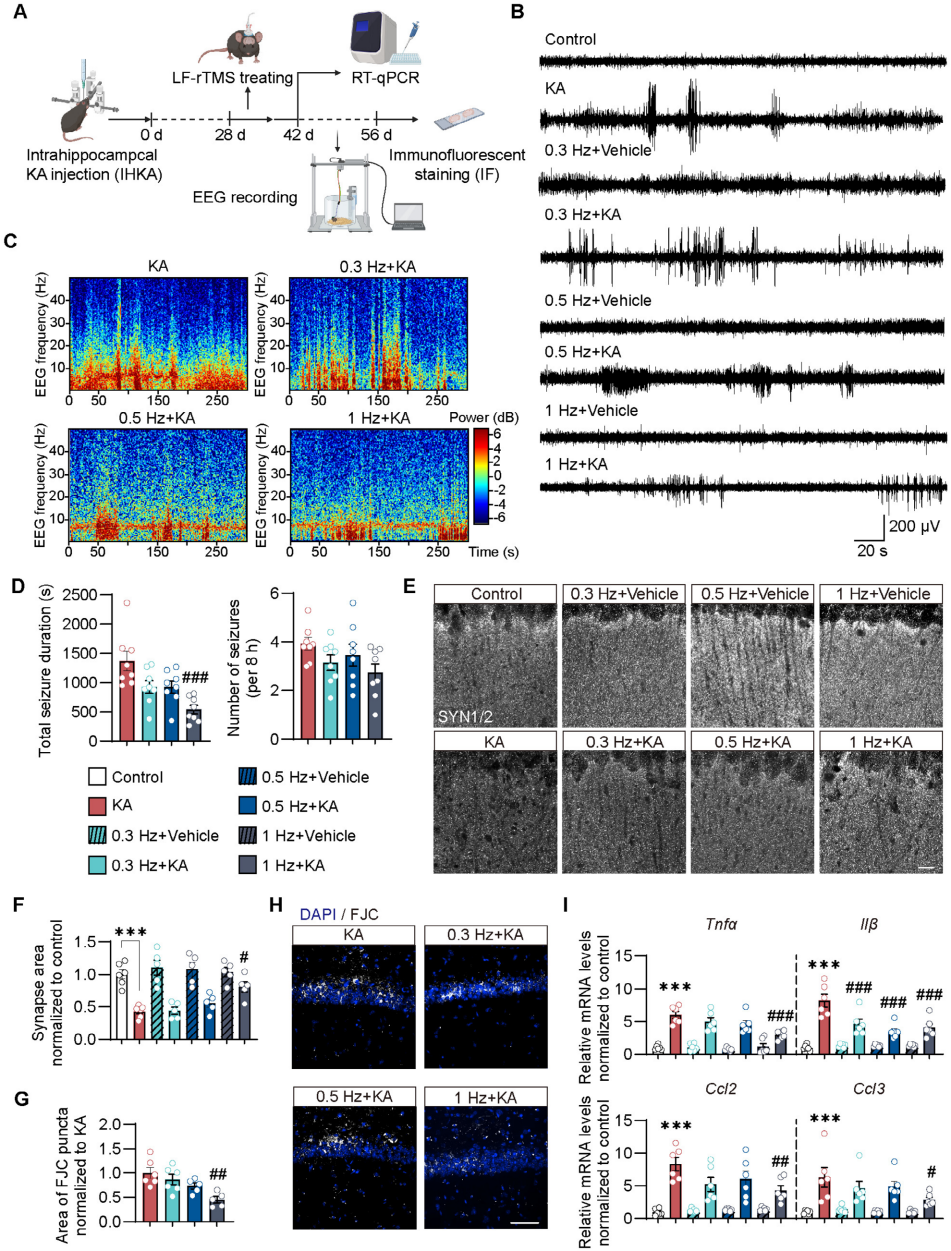
1 Hz LF‐rTMS treatment attenuates epilepsy and reduces transcription levels of inflammatory cytokines in the hippocampus of KA‐induced SE mice. (A) Experimental design schematic. (B, C) Five‐minutes EEG samples display seizure‐like activity in each group. (D) Frequency and total seizure duration analysis confirms that 1 Hz LF‐rTMS (1 Hz + KA) significantly reduces KA‐induced epileptiform discharges. *n* = 8 mice/group. (E) Immunofluorescence images show SYN1/2 (gray) in the hippocampal CA1 stratum radiatum. Scale bar = 100 μm. (F) SYN1/2^+^ area quantification indicates 1 Hz treatment significantly reduces SE‐induced synaptic impairment. (G) Immunofluorescence images show FJC (gray) staining of degenerative neurons in CA1, with DAPI (blue) counterstaining. Scale bar = 100 μm. (H) FJC^+^ area quantification shows 1 Hz treatment significantly reduces SE‐induced neuronal degeneration. (I) qRT‐PCR reveals 1 Hz treatment significantly lowers inflammatory and immunological gene expression in the hippocampus compared to the KA group. *n* = 5–6 mice/group. Data are shown as the means ± SEM. *p < 0.05, **p < 0.01, ***p < 0.001, compared to Control; ^##^ *p* < 0.01, and ^###^ *p* < 0.001. Compared to KA, one‐way ANOVA followed by Tukey's post hoc tests. EEG, electroencephalogram; FJC, Fluoro‐Jade C; IHKA, intrahippocampal KA injection; KA, kainic acid; SE, spontaneous epilepsy; Synapsin1/2, SYN1/2.

We created a chronic SE mouse model by injecting 0.3 μg of KA into the hippocampus, effectively mimicking HS [28, 29, 30]. This model was used to assess the preventive effects of LF‐rTMS on epileptogenesis 28 days post‐KA administration. LF‐rTMS at 0.3, 0.5, and 1 Hz was applied starting on Day 28 (Figure 1A). EEG recordings began on Day 42, showing that 1 Hz LF‐rTMS significantly reduced seizure frequency (Figure 1B–D). Synapsin 1/2 (SYN1/2) and Fluoro‐Jade C staining in the hippocampal CA1 region revealed that 1 Hz stimulation notably reduced neuronal and synaptic loss due to epilepsy (Figure 1E–H). Glial cells in the hippocampus play a key role in triggering and sustaining TLE [10, 28]. Given LF‐rTMS's potential anti‐inflammatory effects [16, 31], we examined inflammatory cytokine transcription levels to assess TLE severity post‐treatment. Our findings suggest that 1 Hz LF‐rTMS reduces epilepsy and neuroinflammation in KA‐induced SE mice (Figure 1I).

### 2.2 1 Hz LF‐rTMS Suppresses Microglial Pathological Activation, Synaptic Phagocytosis, and Epilepsy‐Associated Memory Impairment

**FIGURE 2:**
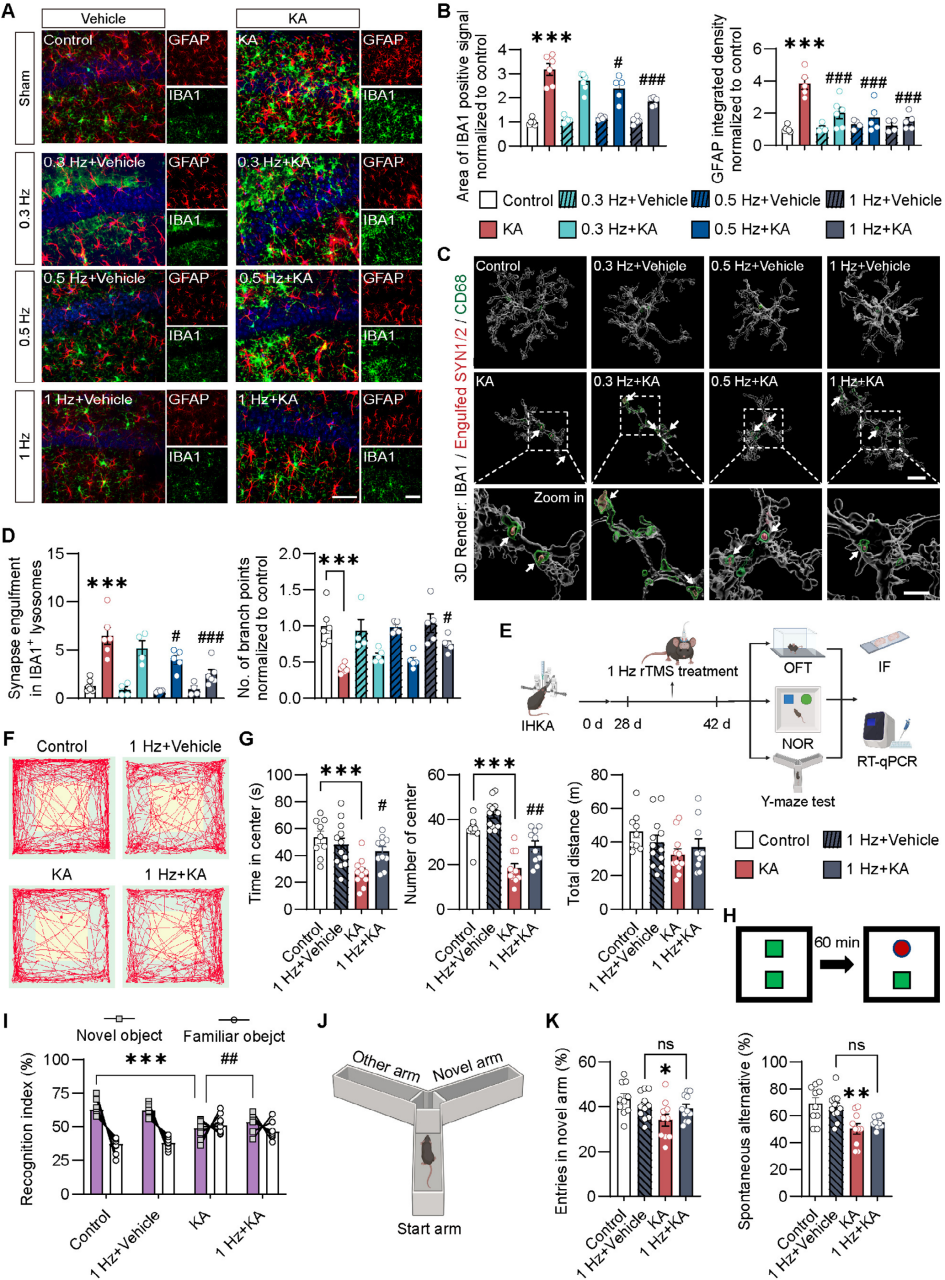
1 Hz LF‐rTMS treatment suppresses microglia‐mediated synaptic phagocytosis and mitigates epilepsy‐associated memory impairment in mice. (A) Immunofluorescence images display GFAP (red) and IBA1 (green) in the hippocampus's CA1 region, with DAPI (blue) marking nuclei. Scale bar = 100 μm. (B) Analysis shows 1 Hz treatment reduces SE‐induced increases in IBA1^+^ glial cells and GFAP^+^ intensity in the CA1 region. *n* = 4–6 mice/group. (C) Immunofluorescence reveals synapses engulfed by microglia in the CA1 region, with CD68 (green), Synapsin 1/2 (red), and IBA1 (white) labeling. Scale bar = 10 μm. (D) Left: 1 Hz LF‐rTMS significantly reduces SE‐induced synaptic phagocytosis by microglia, showing the greatest effect among frequencies. Right: 1 Hz treatment significantly reduces microglia activation compared to the KA group. *n* = 4–6 mice/group. (E) Experimental design for memory tests in mice. *n* = 9–11/group. (F) OFT behavior track for four groups. (G) 1 Hz treatment reduces SE‐induced anxiety‐like behaviors without affecting locomotion, shown by central zone time and total distance. (H‐I) NOR test shows 1 Hz treatment enhances novel object recognition after a 60 min interval. (J) Y‐maze novel arm test. (K) Analysis of novel arm entries and spontaneous alternation rate after free exploration. Data are shown as the means ± SEM. *p < 0.05, **p < 0.01, ***p < 0.001, compared to Control; ^#^ *p* < 0.05, ^##^ *p* < 0.01, and ^###^ *p* < 0.001. Compared to KA; ^ns^ *p* > 0.05, compared to1 Hz + Vehicle. Statistical analyses were performed by one‐way ANOVA with Tukey's post hoc test (B, D, G, K) and two‐way mixed‐effects ANOVA with Tukey's post hoc test (I). KA, kainic acid; NOR, novel object recognition test; OFT, open field test; SE, spontaneous epilepsy.

The contralateral area is most susceptible to secondary epileptogenesis in TLE [30, 32, 33]. Immunofluorescence staining of the contralateral hippocampus in mice revealed microglial and astrocyte marker genes (Figure 2A). Quantitative analysis showed that 0.5 and 1 Hz LF‐rTMS significantly reduced the GFAP signal and IBA1 area in the hippocampal CA1 compared to the KA group (Figure 2B). Neuroinflammation‐induced microglial activation enhances synaptic phagocytosis, disrupting neural circuit homeostasis and contributing to epileptogenesis [8, 10, 11]. To assess LF‐rTMS's impact on microglial phenotypes, a triple‐labeling approach was used with IBA1, ‐SYN1/2, and CD68 to visualize synaptic engulfment by microglia in the hippocampal CA1 (Figure 2C). Quantitative analysis showed a significantly reduced Synapsin1/2 signal in microglia within the hippocampus after 0.5 and 1 Hz LF‐rTMS treatment (Figure 2D). Furthermore, 1 Hz treatment prevented the change of microglia from a resting, branched form to an active, bushy form typically caused by epileptogenesis (Figure 2D). Among the treatment conditions, 1 Hz + KA was the most effective for spontaneous epilepsy in the hippocampus.

To assess the impact of 1 Hz LF‐rTMS on epilepsy‐related memory deficits, we evaluated hippocampal‐dependent short‐term learning and memory. Given that rTMS effects fade posttreatment in TLE, we conducted the following tests: open‐field test (OFT), novel object recognition (NOR), and Y‐maze (Figure 2E). The 1 Hz LF‐rTMS showed an anxiolytic effect without affecting movement (OFT results) (Figure 2F,G). The NOR test indicated improved spatial memory, with a better recognition index than the KA group (Figure 2H,I). In the Y‐maze test, the KA group exhibited significantly fewer novel arm entries and lower spontaneous alternation rates relative to both the Control group and the 1 Hz + Vehicle group (Figure 2J,K). No significant difference was detected between the KA group and the 1 Hz + KA group, and there was no statistical difference between the 1 Hz + KA group and the 1 Hz + Vehicle group (Figure 2J,K), indicating that 1 Hz‐rTMS treatment showed a trend toward improving hippocampal working memory toward the level of the 1 Hz + Vehicle group. Collectively, these findings suggest that 1 Hz LF‐rTMS alleviated the deficits in short‐term memory induced by KA‐induced SE.

### 2.3 Single‐Cell RNA Sequencing Identifies an IFN‐γ‐Responsive DAM State Subsequent to 1 Hz LF‐rTMS

Public bulk RNA sequencing [34] revealed no significant changes in normal hippocampal tissues after a 1 Hz intervention (Figure S1A,B). To explore the impact of 1 Hz LF‐rTMS on microglial synaptic phagocytosis related to epilepsy‐induced memory issues, we performed 10× single‐cell RNA sequencing on 29,771 cells from Control, KA, and 1 Hz + KA groups (Figure S1C–E). Eight cell types were identified via t‐SNE clustering, with microglia being the most prevalent (61.6%) (Figure S1F). Differentially expressed genes (DEGs) were analyzed for each cluster [35], confirming cell type identification (Figure S1G). Significant transcriptional changes were observed, especially in microglia, with 1703 DEGs in KA vs. Control and 417 DEGs in 1 Hz + KA versus KA (Figure S1H).

**FIGURE 3:**
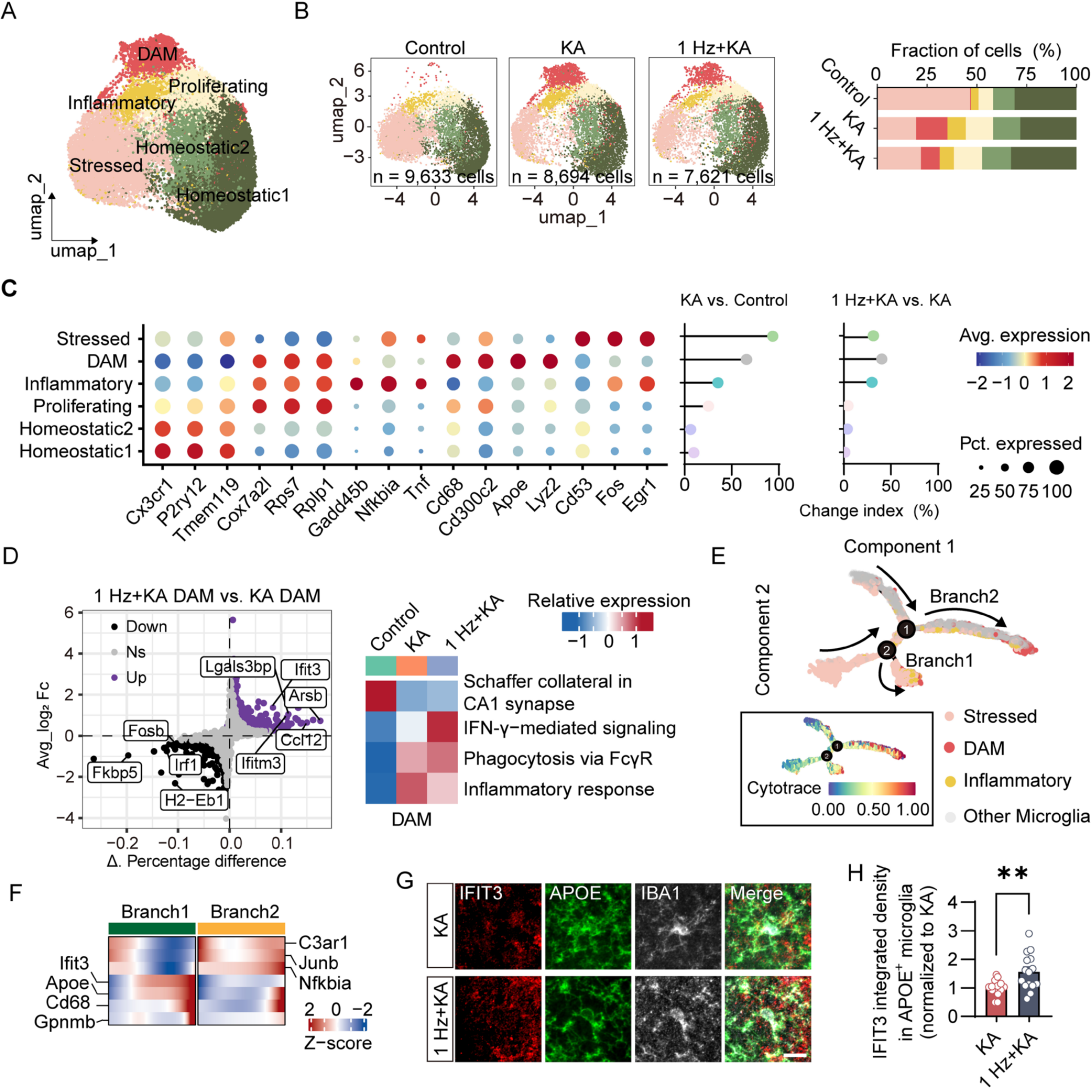
The interferon‐responsive state is heightened in DAM in the mouse hippocampus after 1 Hz LF‐rTMS treatment. (A) Subclustering and UMAP of microglia only. (B) Microglial UMAP visualization showing cell distributions across three groups and stacked barplot depicting the proportions of microglial subsets in the reference atlas from each group. (C) Left: Bubble plot illustrates that microglia are divided into six subsets based on dominant gene expression. Right: Change index from cell type prioritization analysis (CTPA) for each microglial cluster (KA vs. Control; 1 Hz + KA vs. KA) was determined by calculating the absolute difference in proportions divided by their sum. (D) Volcano plot of DEGs in DAM (1 Hz + KA vs. KA group), paired with GO enrichment analysis of these DEGs. Positive zScore values indicate pathway enrichment is enhanced in the KA group. (E) Pseudotime trajectory of microglia is generated, with subsets colored (Stressed: Pink; DAM: Red; Inflammatory: Yellow; others: Gray). DAM shows two differentiation paths (Branch 1/2). Pseudotime is shown with a color gradient by Cytotrace. (F) Heatmap showing expression of key genes in DAM's two differentiation branches (Branch1: Green; Branch2: Orange). (G) Representative IFIT3 (red), APOE (green), IBA1 (gray) triple immunostaining indicates 1 Hz treatment enhances the expression of IFIT3 in the CA1 region of hippocampal microglia. Scale bar = 10 μm. (H) Quantification of IFIT3 signal intensity within APOE^+^ microglia are shown. *n* = 3 images from 5 mice/group. Data are shown as the means ± SEM. **p < 0.01, compared to KA, unpaired Student's *t*‐test. Avg. −log_2_Fc, Average −log_2_ Fold Change; DAM, disease‐associated microglia; Down, downregulated genes; KA, kainic acid; Ns, nonsignificant genes; Up, upregulated genes. Δ. Percentage difference, the difference in a gene's proportion between two groups.

To assess microglial phenotypes before and after 1 Hz LF‐rTMS, we analyzed microglial subsets in the Control group at baseline, KA, and under 1 Hz conditions (Figure 3A,B). We identified six subpopulations, with phagocytic‐associated microglia (DAM or MGnD) [8, 27] showing significant post‐treatment changes (Figure 3C). Gene ontology analysis revealed increased IFN‐γ signaling and synapse loss in DAM (Figure 3 and S2A). Trajectory analysis identified two DAM‐associated branches: one linked to IFNsignaling [36] and another to inflammatory phagocytosis influenced by Nf‐κb [37] and C3ar1 [8] (Figure 3E,F and S2B,C). These findings suggest IFN‐γ signaling's role in microglial phagocytosis in the hippocampus post‐1 Hz treatment, independent of the complement system. While IFN‐γ's role in brain injury and neurodegenerative diseases is known [36, 38], its function in epilepsy is unclear. This study explores its connection to the therapeutic effects of 1 Hz LF‐rTMS in epilepsy. Immunostaining confirmed IFIT3 and APOE co‐expression in IBA1^+^ microglia (Figure 3G,H), supporting our single‐cell findings.

### 2.4 1 Hz LF‐rTMS Alters STAT2 and CADM1/2/3 Expression in Phagocytosing DAM in KA‐Induced Epileptic Mice

**FIGURE 4:**
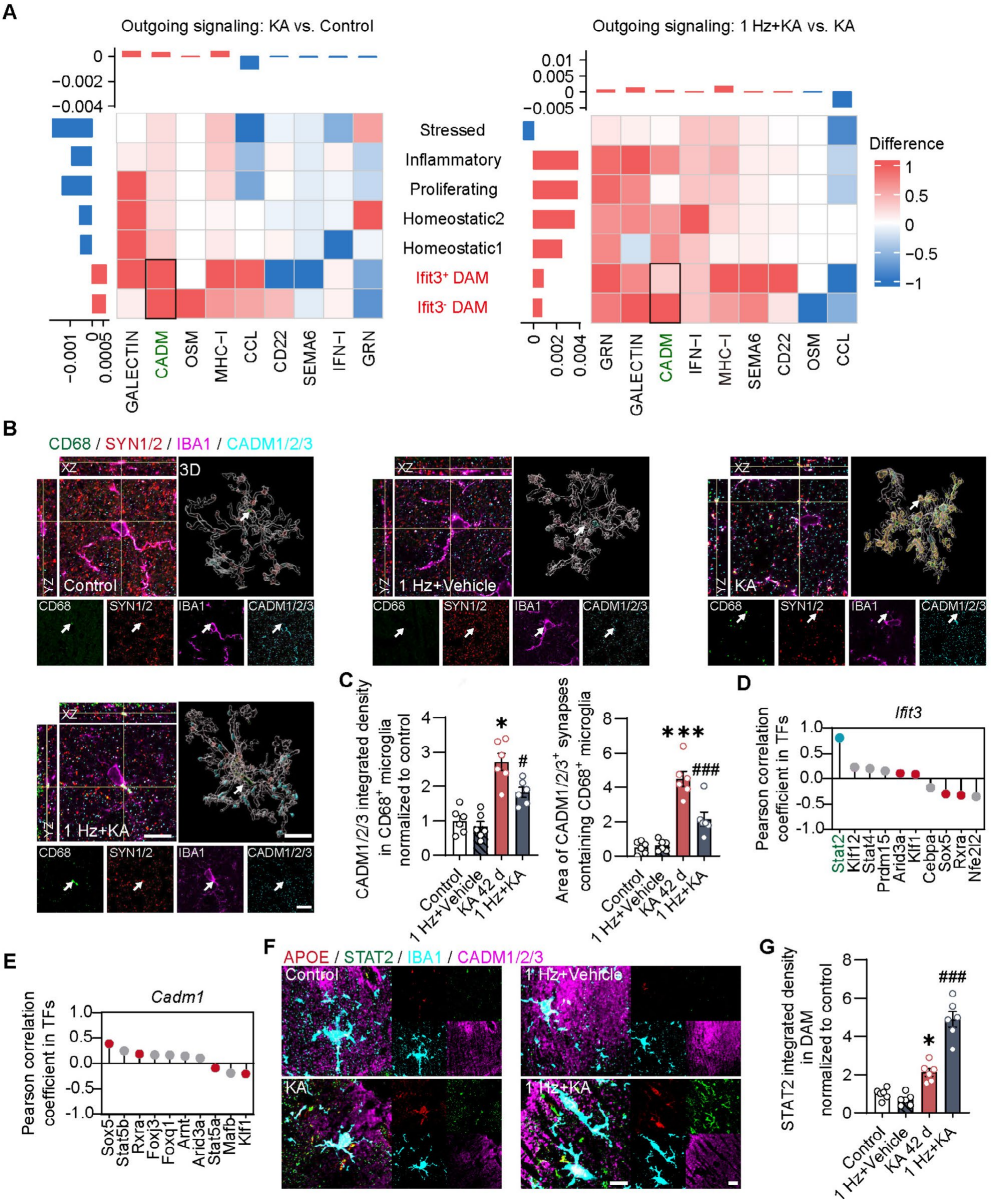
IFN‐γ signaling may regulate CADM1/2/3 expression to ameliorate synaptic phagocytosis in DAM after 1 Hz LF‐rTMS treatment. (A) Heatmap illustrates key ligand‐receptor pathways involved in communication changes pre‐ and post‐1 Hz LF‐rTMS, as identified by Cellchat. (B) Triple staining of SYN1/2 (red), CADM1/2/3 (green), and IBA1 (white) in CA1 hippocampal microglia, scale bar = 10 μm. (C) Left: CADM1/2/3^+^ signal intensity in CD68^+^ microglia quantified. Right: Analysis of synapse engulfment by microglia via CADM1/2/3, *n* = 6 mice/group. (D, E) Lollipop plot of JASPAR‐derived regulons for *Ifit3* and *Cadm1* in microglia. (F) Quadruple staining of STAT2 (green), CADM1/2/3 (magenta), APOE (red), and IBA1 (cyan) in CA1 hippocampal microglia, scale bar = 10 μm. (G) STAT2 signal intensity in DAM quantified, *n* = 6 mice/group. *p < 0.05, **p < 0.01, ***p < 0.001, compared to Control; ^##^ *p* < 0.01, and ^###^ *p* < 0.001. Compared to KA; one‐way ANOVA followed by Tukey's post hoc tests. DAM, disease‐associated microglia; KA, kainic acid.

We investigated the impact of 1 Hz LF‐rTMS on IFN‐γ signaling in phagocytosing DAM in the CA1 hippocampus of a mouse model with KA‐induced chronic SE epilepsy. Ifit3 emerged as one of the most prominent genes in the DAM‐like interferon branch (Figure 3E–H). To clarify its biological interpretation, we emphasize that Ifit3 is viewed as a robust marker of an IFN‐responsive DAM‐like microglial state. The CellChat database showed an upregulation of the CADM pathway in Ifit3^−^ DAM posttreatment (Figures 4A and S2D), indicating that IFN‐γ might inhibit this pathway. CADM1/2/3, usually found in synapses and neurons [39], were present in synaptic phagocytic microglia under pathological conditions [40, 41]. We propose that CADM1/2/3 aid microglia‐mediated phagocytosis. After 1 Hz treatment, synapse loss in the hippocampal CA1 region decreased (Figure S3A–C), with CADM1/2/3 primarily affecting the presynaptic membrane (Figure S3D–H). We analyzed the overlap of CADM1/2/3, SYN1/2, and IBA1^+^ microglia signals in the hippocampal CA1 region to clarify the IFN‐γ‐responsive DAM phenotype (Figure 4B). In the 1 Hz + KA group, CADM1/2/3 signals in IBA1^+^ microglia were weak, with limited overlap with SYN1/2^+^ synapses (Figures 3D and 4B). The area of SYN1/2^+^ synapses co‐localizing with CADM1/2/3 in IBA1^+^ microglia decreased after 1 Hz LF‐rTMS treatment (Figure 4C). Bioinformatic analysis of transcription factor (TF) correlations identified STAT2 as a crucial regulator that exhibits a strong positive correlation with the expression of Ifit3 in the IFN‐γ‐responsive DAM phenotype (Figure 4D). Conversely, Stat2 did not demonstrate a significant positive correlation with Cadm1 transcription in microglia (Figure 4E), and the transcriptional programs associated with Cadm1 showed an overall inverse relationship with those linked to Ifit3. Given these distinct regulatory profiles, we conducted immunostaining to further elucidate the interactions between STAT2, IFIT3, and CADM1/2/3 in DAM. 1 Hz treatment elevated STAT2 fluorescence in CADM1/2/3^+^ DAM cells compared to the KAgroup (Figure 4F,G), highlighting the IFN‐γ pathway's role in DAM functional changes from 1 Hz LF‐rTMS.

### 2.5 Targeting IFN‐γ Signaling May Abrogate the Beneficial Effect of 1 Hz LF‐rTMS Mediated by Microglia‐Mediated Synaptic Phagocytosis

**FIGURE 5:**
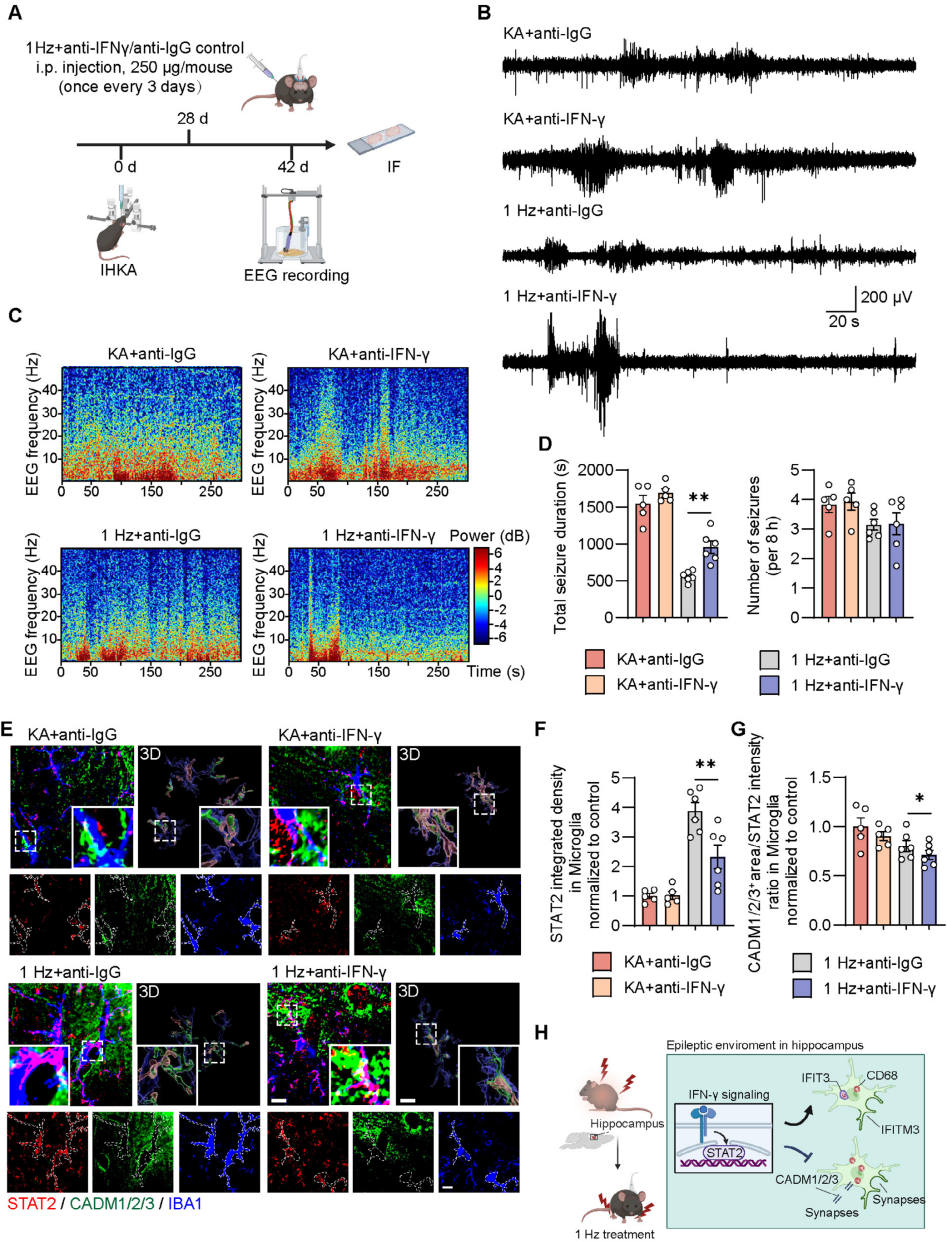
Inhibiting IFN‐γ signaling might negate the benefits of microglia‐mediated 1 Hz LF‐rTMS. (A) Experimental design schematic: (B, C) Five‐minute EEG recordings show seizure‐like activity in each group. (D) Blocking IFN‐γ signaling may negate the suppression of KA‐induced SE discharges by 1 Hz LF‐rTMS, as shown by frequency and seizure duration analysis. *n* = 5–6 mice/group. (E) Representative triple‐labeling of STAT2 (red), CADM1/2/3 (green), and IBA1 (blue) in microglia is shown, 3D reconstructions of microglia are provided for selected regions, scale bar = 10 μm. (F) Quantification of STAT2 signal intensity in microglia. (G) Quantification of the relative ratio of CADM1/2/3^+^ area to STAT2^+^ intensity in microglia. (H) The diagram illustrates that 1 Hz LF‐rTMS treatment in the mouse hippocampus alleviates epilepsy by modulating DAM through the IFN‐γ pathway: Stat2 boosts DAM's IFN‐γ sensitivity and decreases synaptic phagocytosis via CADM1/2/3. Data are shown as the means ± SEM. *p < 0.05, **p < 0.01, ***p < 0.001, compared to 1 Hz + anti‐IgG, one‐way ANOVA followed by Tukey's post hoc tests. EEG, electroencephalogram; KA, kainic acid; SE, spontaneous epilepsy.

To investigate the role of IFN‐γ signaling in the effects of 1 Hz LF‐rTMS on epilepsy and synaptic phagocytosis, we administered an anti‐IFN‐γ antibody or IgG control intraperitoneally (Figure 5A). Blocking IFN‐γ negated the protective effects of 1 Hz LF‐rTMS on epilepsy, increasing seizure duration compared to the control group (Figure 5B–D). Anti‐IFN‐γ also reduced IFIT3 expression in APOE^+^ microglia in the hippocampus (Figure S4A,B), indicating that IFN‐γ blocking alone doesn't affect microglial function without external stimulation [36, 42, 43] like 1 Hz LF‐rTMS. The treatment blocked microglial synaptic engulfment (Figure S4C,D) and reduced STAT2 and CADM1/2/3 expression in microglia following KA‐induced epilepsy (Figure 5E–G). This indicates that IFN‐γ signaling, activated by 1 Hz LF‐rTMS, might regulate microglial synaptic phagocytosis to alleviate epilepsy (Figure 5H).

### 2.6 SnRNA‐Seq Reveals That IFN‐γ Signaling Is Activated in DAM‐Like Phagocytic Microglia Derived From Patients With HS‐Related Epilepsy

**FIGURE 6:**
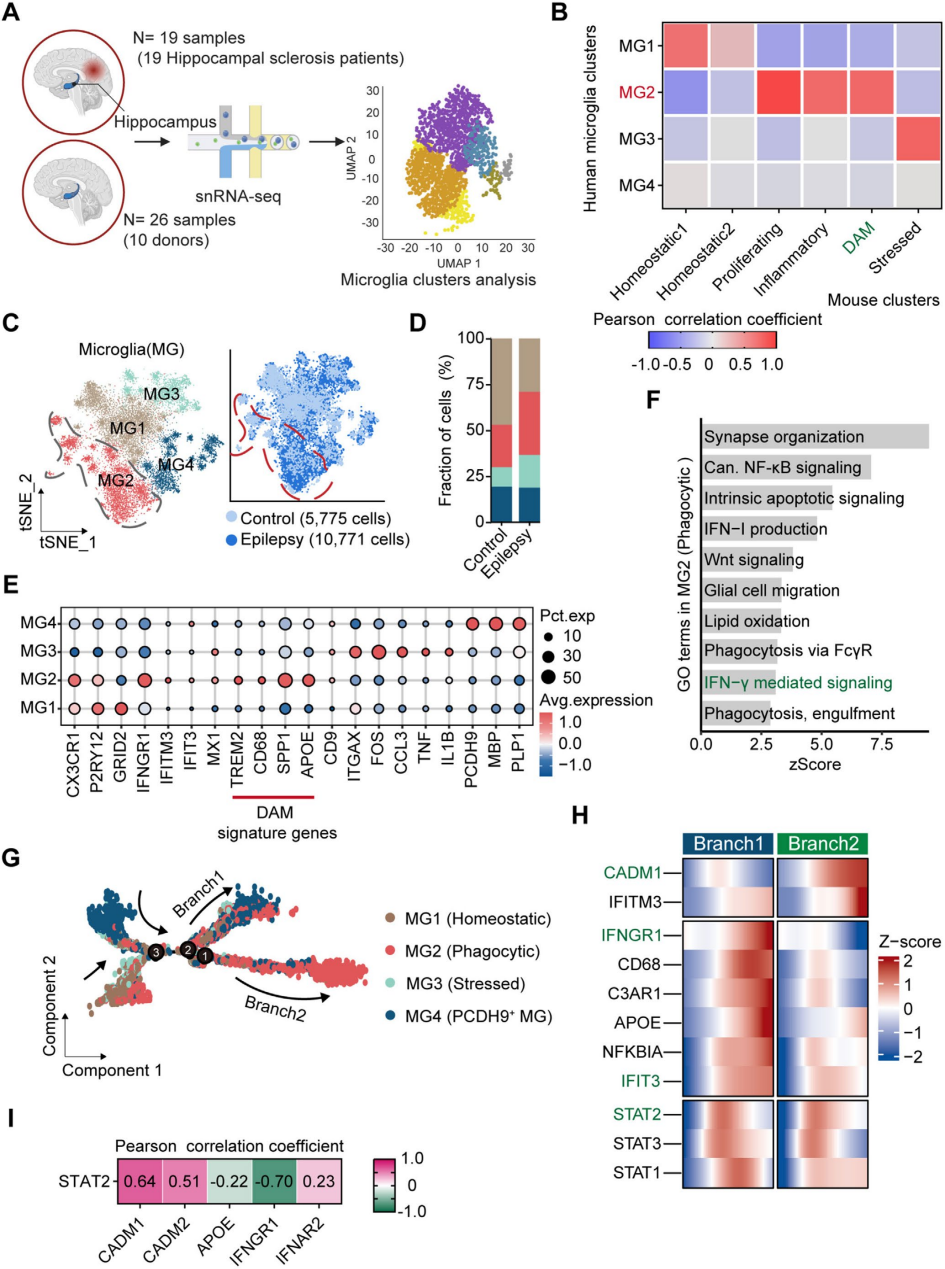
SnRNA‐seq revealed upregulation of IFN‐γ signaling in microglia from patients with HS‐related epilepsy. (A) Schematic of snRNA‐seq using hippocampus samples from HS‐related epileptic participants (*N* = 19 snRNA‐seq libraries) and non‐epileptic controls (*N* = 26 snRNA‐seq libraries from 10 donors). The snRNA sequencing data are available in the GEO database. (B) DAM‐like cells (MG2) found in HS‐related epileptic human hippocampus (C) Subclustering and t‐SNE of microglia only. 5775 and 10,771 sNuc‐seq profiles of human microglia from the hippocampi of nonepileptic individuals (Control) and those with HS‐induced epilepsy (Epilepsy). (D) Stacked barplot depicting the proportions of microglial subsets in the reference atlas from each group. (E) Bubble plot illustrates that microglia are divided into four subsets based on dominant gene expression. (F) GO enrichment analysis of the MG2 subset (Epilepsy vs. Control) identifies it as a phagocytic‐like microglial subset and reveals upregulated IFN‐γ‐mediated signaling in the hippocampi of patients with epilepsy. (G) Pseudotime trajectory of microglia is generated. MG2 (red) shows two differentiation paths (Branch 1/2) that are consistent with mouse scRNA‐seq data. (H) Heatmap showing expression of key genes in MG2's two differentiation branches (Branch1: Blue; Branch2: Green). (I) Heatmap showing Pearson correlation coefficients between STAT2 and selected genes in human MG2 microglia, revealing positive correlations with CADM1 and CADM2, and a negative correlation with IFNGR1. The color gradient indicates correlation strength.

IFN‐γ may oppose IL‐10 in CD68^+^ microglia, affecting CXCR4 and CCR5 in the CA1 region of the hippocampus in human TLE [44]. To explore IFN‐γ's role in 1 Hz LF‐rTMS therapy for HS‐related TLE, we examined DAM or DAM‐like cells in HS tissues from patients [8]. Analysis of 10× snRNA‐seq data from human hippocampi showed comparable microglial populations between epileptic and non‐epileptic individuals (Figure 6A), with a higher proportion of DAM‐like cells in HS‐related epilepsy (Figure 6B). Four microglia subsets were identified (Figure 6C–E): “MG1” with high P2RY12 and CX3CR1, “MG2” with DAM and IFN‐responsive genes, “MG3” with inflammatory and ribosomal genes, and “MG4” with high PCDH9 expression. GO enrichment analysis showed MG2 had a strong IFN‐γ response and changes in phagocytosis‐related processes, aligning with DAM function in mice (Figure 6F). Monocle2 trajectory analysis revealed two DAM‐related phagocytic pathways, with IFNGR1^+^ DAMs rarely co‐localizing with phagocytic genes like *CD68* and *C3AR1* (Figure 6G,H). *STAT2* was also found to upregulate CADM1 in MG2 (Figure 6I). These findings indicate that IFN‐γ signaling and CADM1 in phagocytic microglia play a role in HS‐related epilepsy progression. Overall, snRNA‐seq of the human hippocampus underscores the therapeutic potential of LF‐rTMS and IFN‐γ for epilepsy.

## 3 Discussion

This study demonstrates that 1 Hz LF‐rTMS, but not 0.3 or 0.5 Hz stimulation, effectively alleviates seizures, neuroinflammation, microglia‐mediated synaptic engulfment, and memory impairment in KA‐induced TLE mice. These protective effects were associated with enhanced hippocampal IFN‐γ signaling, consistent with the upregulation of IFN‐γ‐related programs in DAM‐like microglia identified in human HS‐associated epilepsy. Together, these findings suggest that 1 Hz LF‐rTMS may exert therapeutic effects by engaging an IFN‐γ‐associated microglial program, accompanied by changes in STAT2 and CADM1/2/3 expression, ultimately reducing presynaptic engulfment. These results also provide new insight into how electromagnetic stimulation may modulate neuroinflammation and synaptic homeostasis.

Our study provides guidelines for LF‐rTMS in drug‐resistant TLE. rTMS efficacy is frequency‐dependent [26, 45]: high‐frequency worsens seizures, while LF‐rTMS (≤ 1 Hz) offers neuroprotection [8, 21, 26, 45]. Specifically, 0.3 Hz rTMS mitigates drug‐induced epileptogenesis [19], and 0.5–1 Hz frequencies prevent convulsive seizures in rodent models [17, 22]. These results align with studies showing 1 Hz rTMS impacts the CA3‐CA1 hippocampal circuitry via mechanisms similar to long‐term depression [45, 46]. The IHKA mouse model of TLE mimics human pathology, aiding research into mechanisms and treatments. Our study shows that 1 Hz LF‐rTMS significantly reduces chronic seizures, anxiety, and memory issues in this model, with effects similar to clinical results [47]. However, no significant differences were found in the Y‐maze test compared to the KA group (Figure 2K), possibly due to a small sample size or reduced rTMS effects after stopping the intervention.

Our scRNA‐seq data suggest that 1 Hz LF‐rTMS is associated with activation of an IFN‐γ‐responsive microglial state, accompanied by reduced phagocytosis‐related programs after treatment. This interpretation is consistent with previous studies identifying IFIT3^+^ IFN‐responsive DAM‐like microglia with reduced expression of several phagocytosis‐related genes, including APOE, TREM2, SPP1, CLEC7A, and CD68 [36, 38, 48], although the molecular basis by which this state may limit excessive synaptic phagocytosis remains unclear. Accumulating evidence suggests that rTMS may exert therapeutic effects, at least in part, through modulation of synaptic function and microglial activity [49, 50, 51]. Based on this framework, we examined CADM signaling as a candidate pathway associated with the phagocytosis‐suppressed state observed after 1 Hz LF‐rTMS. Our data suggest that 1 Hz LF‐rTMS is accompanied by reduced CADM1/2/3 expression in DAM, together with reduced excessive synaptic phagocytosis. In parallel, STAT2 was increased in association with this IFN‐γ‐responsive microglial state. These findings support a working model in which IFN‐γ/STAT2‐related signaling is associated with CADM1/2/3 downregulation and altered synaptic engulfment after LF‐rTMS, although a direct linear molecular pathway has not yet been established.

To further examine the involvement of IFN‐γ signaling in this process, we used functional IFN‐γ neutralization rather than genetic deletion. Germline or postnatal ablation of microglial IFN‐γ receptors could confound the assessment of LF‐rTMS‐dependent microglial responses; therefore, we administered an IFN‐γ‐neutralizing antibody during rTMS treatment, an approach previously used in acute stress [52], epilepsy [53], and Alzheimer's disease models [36]. Notably, IFN‐γ neutralization attenuated the LF‐rTMS‐associated changes in STAT2 and CADM1/2/3, supporting the interpretation that IFN‐γ signaling contributes to the effects of LF‐rTMS in a context‐dependent manner, rather than acting as a sufficient standalone regulator.

This framework may also be relevant to human disease [38, 44, 48], although the translational connection should be interpreted cautiously. Human snRNA‐seq data suggested increased IFN‐γ‐related microglial signaling in HS‐related epilepsy, supporting the relevance of this pathway across species. In addition, previous work has shown that STAT2 contributes to interferon responsiveness in human iPSC‐derived microglia [48], while Cadm1 upregulation has been reported in human hippocampal DAM [54]. Together, these observations support the possibility that CADM‐associated synaptic pathology may represent a therapeutically relevant downstream pathway, but its precise regulation by IFN‐γ/STAT2 signaling will require further mechanistic investigation.

Our study has limitations, including the use of only one scRNA‐seq library per group, which may introduce bias. We minimized the control group of healthy mouse hippocampi due to their similarity to normal mice in bulk RNA‐seq. Because our scRNA‐seq analysis was performed on dissociated whole‐cell suspensions, the relative enrichment of microglia in the dataset should be interpreted with caution and not as a direct measure of in situ abundance. In the IFN‐γ blocking assay, we focused on key contrasts by reducing healthy mouse intervention groups. We did not conduct the Morris water maze and conditioned fear tests because 1 Hz LF‐rTMS might be ineffective, and epilepsy affected the amygdala. Our study only partially explored the interaction between adaptive immunity and microglia before and after 1 Hz rTMS treatment. Although we used a neutralizing antibody to inhibit peripheral IFN‐γ, further depletion in T cells could clarify peripheral adaptive immunity's role in epilepsy.

In summary, 1 Hz LF‐rTMS modulates hippocampal microglial phagocytic specificity via enhanced IFN‐γ signaling, shifting microglia from synaptic elimination to preservation, offering new options for DRE and synaptic loss conditions.

## 4 Materials and Methods

### 4.1 Animals

Eight‐week‐old male C57BL/6J mice, each weighing 20 ± 2 g, were sourced from Vital River Laboratory Animals, Beijing, China. They were kept at 22°C–25°C, 50%–60% humidity, with a 12‐h light/dark cycle, and had food access. All procedures followed the guidelines of the Animal Laboratory Center of Pediatrics, Children's Hospital of Chongqing Medical University. Only male mice were used in this study to minimize potential variability associated with the estrous cycle in female mice and to maintain experimental consistency, as reported in previous studies [55, 56].

### 4.2 KA Injection the CA1 Region of the Hippocampus

KA (0.6 μg/μL) in artificial cerebrospinal fluid (ACSF) was injected into the right hippocampus of mice using a stereotaxic apparatus at coordinates: A/P −1.6 mm, M/L + 2.03 mm, D/V −1.5 mm. The injection volume was 500 nL for both KA and control groups, with controls receiving only ACSF [57].

### 4.3 LF‐rTMS Treatment

Four weeks after KA or ACSF injection, mice received daily LF‐rTMS for 14 consecutive days [17] using a CCY‐I magnetic stimulator [50] equipped with a 34‐mm circular coil. Stimulation was delivered at 20% of the maximum stimulator output [26] (corresponding to 25% of the device's 1 T maximal magnetic field), without motor‐threshold calibration. Frequencies of 0.3, 0.5, and 1.0 Hz were applied with inter‐block intervals of 1 s for the 0.3‐ and 0.5‐Hz groups and 2 s for the 1.0‐Hz group. Each block contained 6, 7, and 10 pulses, respectively, and was repeated 100, 120, and 125 times, yielding total daily treatment durations of 35, 30 and 25 min. Mice were restrained to maintain consistent coil positioning over the skull, whereas sham‐treated mice underwent identical procedures without active stimulation. See Data S1 for full details.

### 4.4 EEG Recordings

Mice were individually housed in wire cages, with electrodes connected to an EEG system (AR‐4 M, QL, China) to monitor brain activity while allowing free movement and access to food and water. Epileptiform discharges, identified by high‐frequency oscillations lasting 15–30 s and at least double the baseline amplitude [29], were visually reviewed by a neurophysiologist to pinpoint seizure start and end times. The coastline index, measuring the sum of distances between consecutive data points for each seizure EEG, was calculated as per previous studies [58].

### 4.5 Anti‐IFN‐γ Administration

Anti‐IFN‐γ antibody (BE0055, BioXcell, USA) or IgG1 isotype control (BE0088, BioXcell; USA) was administered via intraperitoneal injection (i.p.) at a dose of 250 μg per mouse [36, 52]. The initial injection was given 14 h before starting 1 Hz rTMS treatment, and booster doses of 250 μg were administered every 3 days until the treatment ended to maintain stable antibody levels.

### 4.6 RNA Extraction and qRT‐PCR

RNA was extracted from tissue using TRIzol (R0016, Beyotime, CHN), quantified with a Nanodrop 2000 (Thermo, USA), and reverse‐transcribed with an RT reagent Kit (RR037A, Takara, JPN). Real‐time PCR was performed with SYBR Mix (Q711‐02, Vazyme, CHN) on a CFX96 system, with all samples tested in duplicate. Primer sequences are in Table 1, and results were analyzed using the 2^−ΔΔ*C*t^ method.

**TABLE 1:** Primer sequences.

Gene	Primer	Sequence
*Il‐1β*	Forward	GCAGAGCACAAGCCTGTCTTCC
	Reverse	ACCTGTCTTGGCCGAGGACTAAG
*Tnf‐α*	Forward	ACTCCAGGCGGTGCCTATGT
	Reverse	GTGAGGGTCTGGGCCATAGAA
*Ccl2*	Forward	TTAAAAACCTGGATCGGAACCAA
	Reverse	GCATTAGCTTCAGATTTACGGGT
*Ccl3*	Forward	TTCTCTGTACCATGACACTCTGC
	Reverse	CGTGGAATCTTCCGGCTGTAG
*β‐Actin*	Forward	GACCCAGATCATGTTTGAGA
	Reverse	GAGCATAGCCCTCGTAGAT

### 4.7 Immunohistochemistry and Confocal Microscopy

Mice were deeply anesthetized and transcardially perfused with PBS. Brains were collected, fixed in 4% paraformaldehyde overnight at 4°C, cryoprotected in 30% sucrose, and sectioned coronally at 30 μm [10, 11, 59]. Sections were blocked for 1 h, incubated overnight with primary antibodies, treated with fluorescent secondary antibodies, followed by staining with 0.001% Fluoro‐Jade C Staining Kit (Biosensis, TR‐100‐FJT) for 30 min, then mounted with DAPI Fluoromount‐G (0100–20, SouthernBiotech, USA). Primary antibodies: anti‐IBA1 (PRB029‐01/PGP049‐01, 1:400, Oasis Biofarm, CHN; HA610221, 1:400, Huabio, CHN), anti‐GFAP (AB5541, 1:1000, Millipore, USA), anti‐CD68 (ab955, 1:200, Abcam, UK), antisynapsin1/2 (106004, 1:1000, Synaptic systems, GER), anti‐APOE (sc‐390925, 1:400, Santa Cruz Biotechnology, USA), anti‐IFIT3 (67208‐1‐Ig, 1:300, Proteintech, CHN), anti‐STAT2 (sc‐514193, 1:300, Santa Cruz Biotechnology, USA), anti‐vGLUT1/2 (135503, 1:1000, Synaptic systems, GER), anti‐PSD95 (HA610256, 1:250, Huabio, CHN), anti‐vGAT (HA601471, 1:400, Huabio, CHN), antigephyrin (147111, 1:250, Synaptic systems, GER), anti‐CADM1/2/3 (243003, 1:500, Synaptic systems, GER). Z‐stacks (0.5 μm steps) were captured via CSU‐W1Sora confocal microscope (Nikon, JPN) with a ×60 oil objective, maintaining consistent exposure and laser power. Imaris (Bitplane) was used for 3D surface rendering of microglia (thresholding‐based), and ImageJ (Fiji) analyzed fluorescent co‐localization. See Data S1 for full details.

### 4.8 Behavioral Tests

Mice were acclimated for 5 days to minimize stress before sequential behavioral tests following 1 Hz rTMS treatment. Four groups were tested: Control, 1 Hz + Vehicle, KA, and 1 Hz + KA. Tests were conducted blindly, with apparatuses cleaned between subjects to remove scent cues. Open field test (OFT): Conducted 1 day post‐rTMS in a 40 cm × 40 cm × 30 cm box. Mice explored for 10 min, with movements recorded and analyzed for locomotion and area preference. Novel Object Recognition Test (NORT): Conducted the day after OFT in the same arena. Mice explored one novel and one familiar object for 8 min. Memory was assessed by the recognition index (time spent on the novel object vs. total exploration time). Y‐maze Test: Consisted of three identical arms at 120° angles. Two phases: (1) Training with one arm blocked for 8 min; (2) after a 1‐h interval, the blocked arm was opened for an 8‐min test to measure time spent in the novel arm.

### 4.9 PCA Analysis of Bulk RNA‐Seq Data

Bulk RNA‐seq data of hippocampal tissues from 1 Hz rTMS and Sham groups were retrieved from GEO database (GEO accession: GSE230150) [26]. PCA analysis was performed on *z*‐score transformed using the prcomp function. The first two principal components (PC1/PC2) were visualized via ggplot2 (v3.5.0), with samples colored by group (1 Hz vs. Sham) to assess transcriptomic heterogeneity and group separation.

### 4.10 ScRNA‐Seq Data Collection in Mouse Hippocampus

Three experimental groups were set: Control (ACSF‐injected), KA (KA‐injected, sacrificed 42 days postinjection), and 1 Hz + KA (1 Hz LF‐rTMS for 2 weeks, initiated 28 days post‐KA injection). For each group, contralateral hippocampal tissues from 8 male mice were pooled in tissue preservation solution (130‐100‐008, Miltenyi, GER) before processing. Single‐cell libraries were constructed using Chromium Single Cell 3′ Reagent Kits v3 (10× Genomics). Briefly, cells were washed three times with 0.04% BSA DPBS, resuspended to 700–1200 cells/μL (viability ≥ 85%), and subjected to droplet‐based cell capture, reverse transcription, and cDNA amplification. Libraries were sequenced on an Illumina NovaSeq platform with a PE150 strategy (Chongqing Allbioknow Biotechnology Co. Ltd., CHN).

### 4.11 Celltype Identified

Raw data were imported via Seurat (v5.3.1) *Read10X*. Quality control excluded cells with < 300 features or > 10% mitochondrial genes; doublets were removed using DoubletFinder (v2.0.3, cutoff = 0.075). Data were normalized by SCTransform, top 2000 variable genes identified, and PCA performed. Microglial subclusters were annotated based on cluster‐enriched marker genes identified by Seurat *FindAllMarkers* and their associated GO pathway enrichment, following established principles for microglial state classification in prior scRNA‐seq studies [8, 35].

### 4.12 Differential Expression Gene (DEG) and Pathway Analysis

DEGs were identified via Seurat *FindAllMarker* (Wilcoxon Rank Sum test) with thresholds: |pct.1‐pct.2| > 0.1, log FC ≥ 0.25, *p* < 0.05. Gene set enrichment analysis validated cell type annotation.

### 4.13 Pseudotime Trajectory Analysis

Monocle (v2.38.0) was used for pseudotime analysis, validated by CytoTRACE (v1.0.0). DEGs along pseudotime were filtered by adjusted *p* < 0.05 and |log FC| ≥ 1.0, with dynamics visualized via heatmaps.

### 4.14 Ligand–Receptor (L–R) Analysis

CellChat (v1.6.1) constructed L–R networks using CellChatDB.mouse. Differentially overexpressed genes and L–R pairs (|log FC| ≥ 1.0, *p* < 0.05) were identified; intercellular communication probabilities and pathways were calculated. CellChat objects were aligned via *liftCellChat* before merging.

### 4.15 SnRNA‐Seq Analysis of Human Hippocampal Tissues

Public data (GEO: GSE GSE264624 [41], GSE275302) from normal donors and epilepsy‐induced HS patients were processed with Seurat: cell filtering, CCA for batch correction, PCA (top 2000 variable genes), and Louvain clustering. Microglia (P2RY12^+^/CX3CR1^+^) were further analyzed via SCTransform, CCA, PCA, clustering (resolution = 0.5), t‐SNE, and DEG analysis (*FindAllMarkers*: Wilcoxon test, min.pct = 0.25, |log FC| ≥ 1.0). No written consent has been obtained from the patients as there is no patient‐identifiable data included.

### 4.16 Comparison With Human Single‐Cell Sequencing Datasets

Spearman's rank correlation compared normalized homologous DEG profiles between mouse and human microglial subclusters (Hmisc corr.test, *p* < 0.05), with results visualized as heatmaps.

### 4.17 TF Analysis Integrated With WGCNA

hdWGCNA (v0.4.08) integrated WGCNA: top 2000 variable genes, signed network (soft thresholding power = 14, min.k = 35, 20 modules) to identify Ifit3^−^ specific signatures. JASPAR (v1.40.0) motifs scanned DNA regions; ConstructTFNetwork built TF‐target networks. XGBoost identified TFs predicting target gene expression.

### 4.18 Statistical Analysis

Quantitative results are presented as mean ± SEM from a minimum of three independent experiments. Statistical analysis was conducted using GraphPad Prism 10.0 (GraphPad Software Inc., San Diego, CA, USA). The normality of data distribution was assessed using the Shapiro–Wilk test. Data with a normal distribution were analyzed using parametric tests, including one‐way ANOVA followed by Tukey's post hoc test for multiple group comparisons and an unpaired Student's *t*‐test for single group versus control comparisons. Data that were not normally distributed were analyzed using the nonparametric Kruskal–Wallis test. Two‐way ANOVA was used for the analysis of data in Figure 2I. A *p*‐value of less than 0.05 was considered statistically significant.

## Author Contributions

D.L. and N.X. conceived and designed the study. D.L. and D.W. performed the experiments and analyzed the results. D.L. and N.X. wrote and revised the manuscript. D.L. participated in the data acquisition, analysis, and interpretation. N.X. provided funding support.

## Funding

The authors have nothing to report.

## Ethics Statement

All experimental procedures described in this article were approved by the Ethics Committee of Children's Hospital of Chongqing Medical University (Ethical approval code: CHCMU‐IACUC20250609005).

## Consent

The authors have nothing to report.

## Conflicts of Interest

The authors declare no conflicts of interest.

## Supporting information


**Data S1:** 1 LF‐rTMS system and stimulation protocols.

**Figure S1:** Integrated transcriptomic overview of bulk RNA‐seq and single‐cell RNA‐seq datasets in the rodent hippocampus following 1 Hz LF‐rTMS.
**Figure S2:** 1 Hz LF‐rTMS isoform‐dependent atlas of hippocampal microglia in epilepsy.
**Figure S3:** 1 Hz LF‐rTMS treatment may regulate CADM1/2/3 expression to prevent the loss of excitatory synapses in the hippocampus of epileptic mice.
**Figure S4:** Inhibiting IFN‐γ signaling negates the beneficial effects of 1 Hz LF‐rTMS on microglial synaptic phagocytosis.

## Data Availability

The data that support the findings of this study are available on request from the corresponding author. The data are not publicly available due to privacy or ethical restrictions.
