# Supplementary material for: 1 Hz Low‐Frequency Repetitive Transcranial Magnetic Stimulation Ameliorates Epilepsy by Suppressing Interferon‐γ Signaling‐Dependent Microglial Synaptic Phagocytosis in Mice
Source: CNS Neurosci Ther. 2026 Jun 13;32(6):e70979. doi: 10.1002/cns.70979 (PMC13263791; doi:10.1002/cns.70979)
Supplement: Supplementary file 1 — Data S1: 1 LF‐rTMS system and stimulation protocols. [file CNS-32-e70979-s001.docx]

**Supplementary Materials: Detailed Methodology**

**Corresponding Manuscript Title:** *1 Hz low-frequency repetitive transcranial magnetic stimulation ameliorates epilepsy by suppressing interferon-γ signaling-dependent microglial synaptic phagocytosis in mice*

Authors: Donghui Lin, Duan Wang, Nong Xiao

Journal: CNS Neuroscience & Therapeutics

This supplemental file provides additional methodological details that could not be included in the main manuscript because of word count limitations.

**1. Introduction to Supplemental Methodology**

To fully address the reviewers’ concerns regarding the methodological details, we provide additional information in this supplemental file, including optimized procedures, parameter settings, and experimental details that are essential for the reproducibility of the study but exceed the main manuscript’s word limit.

**2. Detailed Methodology**

**2.1 LF-rTMS Treatment**

Four weeks after KA or ACSF injection, mice underwent daily low-frequency repetitive transcranial magnetic stimulation (LF-rTMS) for 14 consecutive days^1^. Stimulation was delivered using a CCY-I magnetic stimulator (YRD CCY-I, Wuhan Yiruide Medical Equipment Co., Ltd., China) equipped with a circular 34 mm animal research coil (model Y034, Wuhan Yiruide Medical Equipment Co., Ltd., China); the coil was equipped with an inert liquid internal circulation cooling system^2^, with a coil length of 110 mm, coil diameter of 34 mm, and magnetic field change rate of 50 kT/s. The stimulation waveform was biphasic, with a pulse period of 340 μs and pulse width of 120 μs (Fig.S1.1 A). The stimulation intensity was fixed at 20% of the maximum stimulator output (corresponding to 25% of the 1 T maximum magnetic field intensity), as described previously^3^; no motor threshold (MT) calibration was performed, and the fixed intensity approach was adopted. For LF-rTMS protocols, stimulation frequencies of 0.3, 0.5, and 1.0 Hz were applied, with inter-block intervals of 1 s for the 0.3 and 0.5 Hz groups and 2 s for the 1.0 Hz group. Each block contained 6, 7, and 10 pulses, corresponding to block durations of 20, 15, and 10 s, and was repeated 100, 120, and 125 times (Fig.S1.1 B), resulting in total daily treatment times of 35, 30, and 25 min and total daily pulse numbers of 600, 840, and 1250 for the 0.3-, 0.5-, and 1.0-Hz groups, respectively. To ensure standardized coil placement over the skull region corresponding approximately to the hippocampus, mice were restrained in custom-made holders to maintain a consistent posture during stimulation. The coil was placed vertically in close contact with the skull surface. Downstream histological analyses focused on the hippocampal CA1 region. For sham controls, mice underwent identical restraint and coil-positioning procedures, with the stimulator set to 0% output and no active magnetic stimulation delivered.


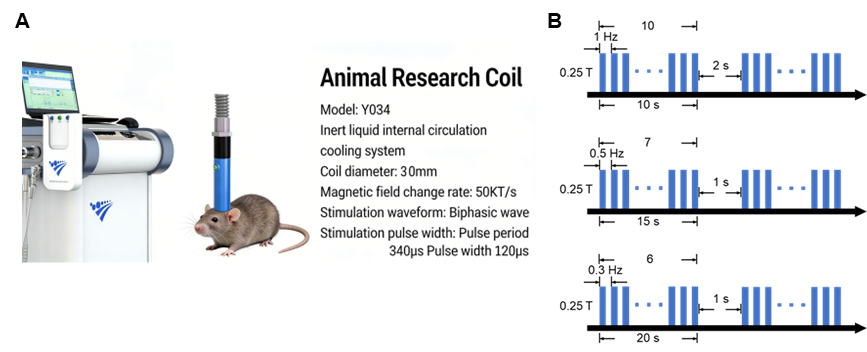


**Fig. S1.1** LF-rTMS system and stimulation protocols. (A) The animal TMS system and coil parameters (model Y034, circular 34 mm, inert liquid cooling, 50 kT/s magnetic field change rate, biphasic waveform with a pulse period of 340 μs and a pulse width of 120 μs). The coil was placed vertically over the skull above the hippocampal region, and downstream analyses focused on CA1. (B) Schematic of the three LF-rTMS protocols (1.0, 0.5, and 0.3 Hz) showing single-pulse timing, pulses per block, inter-block intervals, and block durations. Stimulation intensity was set at 20% of maximum stimulator output (MSO), corresponding to approximately 0.25 T of the device’s 1 T maximal magnetic field, and no motor-threshold calibration was performed.

**2.2 Immunostaining and image analysis**

After deep anesthesia, mice were transcardially perfused with PBS, and brains were extracted, fixed in 4% paraformaldehyde overnight at 4 °C, and then transferred into 30% sucrose solution. Brain sections (30 μm) were cut using a sliding microtome and stored at −20 °C in cryoprotectant. For each animal, 3–4 corresponding hippocampal sections were collected for analysis. After washing in PBS, sections were blocked with QuickBlock™ blocking buffer for immunostaining (P0260, Beyotime, China) for 1 h. No antigen retrieval was performed. Sections were then incubated in primary antibodies diluted in *QuickBlock*™ primary antibody dilution buffer (P0262, Beyotime, China) overnight at 4 °C: anti-IBA1 (1:400, Oasis Biofarm; 1:400, Huabio), anti-GFAP (1:1000, Millipore), anti-CD68 (1:200, Abcam), anti-synapsin1/2 (1:1000, Synaptic Systems), anti-APOE (1:400, Santa Cruz Biotechnology), anti-IFIT3 (1:300, Proteintech), anti-STAT2 (1:300, Santa Cruz Biotechnology), anti-vGLUT1/2 (1:1000, Synaptic Systems), anti-PSD95 (1:250, Huabio), anti-vGAT (1:400, Huabio), anti-gephyrin (1:250, Synaptic Systems), anti-CADM1/2/3 (1:500, Synaptic Systems). After washing, sections were incubated with corresponding Alexa Fluor–conjugated secondary antibodies (P0265, Beyotime, China) diluted in QuickBlock™ secondary antibody dilution buffer for 2 h at 37 °C. Finally, sections were mounted with DAPI Fluoromount-G (0100-20, SouthernBiotech, USA). Z‑stacks (0.5 μm steps) were captured via a CSU‑W1 Sora confocal microscope (Nikon, Japan) equipped with a 60 × oil immersion objective. Three random fields from three hippocampal sections were analyzed per mouse, with background subtraction and uniform thresholding applied to all images for consistency.

**2.3 Microglial morphology and 3D reconstruction**

Z-stack images were acquired with a step size of 0.5 μm using a 60× oil-immersion objective on a CSU-W1 Sora confocal microscope (Nikon), spanning a thickness of 20 μm. For morphological analysis, Z-stack images were Z-projected, binarized, and skeletonized using the Skeletonize3D plugin in Fiji/ImageJ. Distal branches were quantified from the skeletonized images. Representative 3D reconstructions were generated from the original Z-stacks using Imaris software (Bitplane).

**2.4 Fluoro‑Jade C (FJC) staining**

Brain sections were processed for FJC staining either after blocking and three PBS washes (for FJC single staining) or after completion of immunofluorescence staining and three PBS washes following secondary antibody incubation (for FJC co‑staining). Potassium permanganate pretreatment was omitted based on our optimized protocol for compatibility with immunofluorescence co-staining and acceptable signal-to-background performance. Sections were incubated with 0.001% FJC for 30 min and washed with PBS three times before mounting.

**2.5 Synaptic puncta staining and analysis**

To assess microglial phagocytosis of synaptic elements, brain sections were triple-labeled for the lysosomal marker CD68 (phagocytic microglia), presynaptic Synapsin1/2, and the microglial marker IBA1. For quantitative synaptic puncta analysis, sections were additionally labeled for presynaptic markers (vGLUT1/2, vGAT) and postsynaptic markers (PSD95, gephyrin). Quantification was performed using ImageJ with the Puncta Analyzer plugin^4^, which detects and counts pre‑synaptic, post‑synaptic, and colocalized synaptic puncta.

**2.6 Immunofluorescence quantification and colocalization analysis**

Quantitative colocalization analysis between IBA1-positive microglia and target proteins (CADM1/2/3, STAT2, IFIT3, and APOE) was performed using ImageJ software. IBA1-positive microglial ROIs were defined first, and colocalization measurements for target proteins were restricted to these ROIs.

Briefly, acquired composite fluorescent images were first separated into individual grayscale channels using the *Split Channels* tool in the *Image* menu under the *Color* submenu. For each channel, positive signals were segmented using the *Threshold* tool in the *Image* menu under the *Adjust* submenu, with either manual adjustment or Otsu’s automatic thresholding, followed by application to generate binary images. Signal morphology was refined using the *Fill Holes* and *Watershed* functions in the *Process* menu under the *Binary* submenu as needed.

Colocalized signals were identified as overlapping regions using the *ROI Manager* tool in the *Analyze* menu under the *Tools* submenu. Binary masks from target channels were added as separate ROIs, and intersection (*AND*) operations were applied to obtain regions of dual‑positive colocalization. For triple‑positive signals, sequential intersection steps were performed.

The number, area, and mean intensity of colocalized puncta were automatically quantified using the *Analyze Particles* tool in the *Analyze* menu, with edge signals excluded to avoid quantification bias. Uniform thresholding and particle selection criteria were applied across all images to ensure reproducibility.

**3. Cross-Reference to Main Manuscript**

The detailed methods provided above correspond to the following sections of the main manuscript:

- **LF-rTMS Treatment** in the main manuscript → Section 2.1 in this supplemental file
- **Immunohistochemistry and confocal microscopy** in the main manuscript → Section 2.2 - 2.6 in this supplemental file

**4. References**

1. Chen R, Spencer DC, Weston J, Nolan SJ. Transcranial magnetic stimulation for the treatment of epilepsy. *The Cochrane database of systematic reviews.* 2016(8):Cd011025.

2. Huang M, Di J, Li N, et al. Repetitive Trans-spinal Magnetic Stimulation Suppresses Microglia to Engulf Synapse and Promotes Nerve Repairment via cGAS-STING Signaling Pathway after Spinal Cord Injury. *International journal of biological sciences.* 2025;21(14):6132-6155.

3. Kistsen V, Evstigneev V, Dubovik B, Ulashchik V. The Effects of Repetitive Transcranial Magnetic Stimulation on Picrotoxin-Induced Convulsions in Mice. *Adv Clin Exp Med.* 2016;25(2):317-325.

4. Ippolito DM, Eroglu C. Quantifying synapses: an immunocytochemistry-based assay to quantify synapse number. *Journal of visualized experiments : JoVE.* 2010(45).
