# Supplementary material for: 1 Hz Low‐Frequency Repetitive Transcranial Magnetic Stimulation Ameliorates Epilepsy by Suppressing Interferon‐γ Signaling‐Dependent Microglial Synaptic Phagocytosis in Mice
Source: CNS Neurosci Ther. 2026 Jun 13;32(6):e70979. doi: 10.1002/cns.70979 (PMC13263791; doi:10.1002/cns.70979)
Supplement: Supplementary file 2 — Figure S1: Integrated transcriptomic overview of bulk RNA‐seq and single‐cell RNA‐seq datasets in the rodent hippocampus following 1 Hz LF‐rTMS. Figure S2: 1 Hz LF‐rTMS isoform‐dependent atlas of hippocampal microglia in epilepsy. Figure S3: 1 Hz LF‐rTMS treatment may regulate CADM1/2/3 expression to prevent the loss of excitatory synapses in the hippocampus of epileptic mice. Figure S4: Inhibiting IFN‐γ signaling negates the beneficial effects of 1 Hz LF‐rTMS on microglial synaptic phagocytosis. [file CNS-32-e70979-s002.docx]

**Supplementary Figure**

**Corresponding Manuscript Title:** *1 Hz low-frequency repetitive transcranial magnetic stimulation ameliorates epilepsy by suppressing interferon-γ signaling-dependent microglial synaptic phagocytosis in mice*

Authors: Donghui Lin, Duan Wang, Nong Xiao

Journal: CNS Neuroscience & Therapeutics

This file contains supplementary figures that provide additional experimental data supporting the main manuscript findings, which could not be fully included due to space limitations. All supplementary figures are cited in the main text.


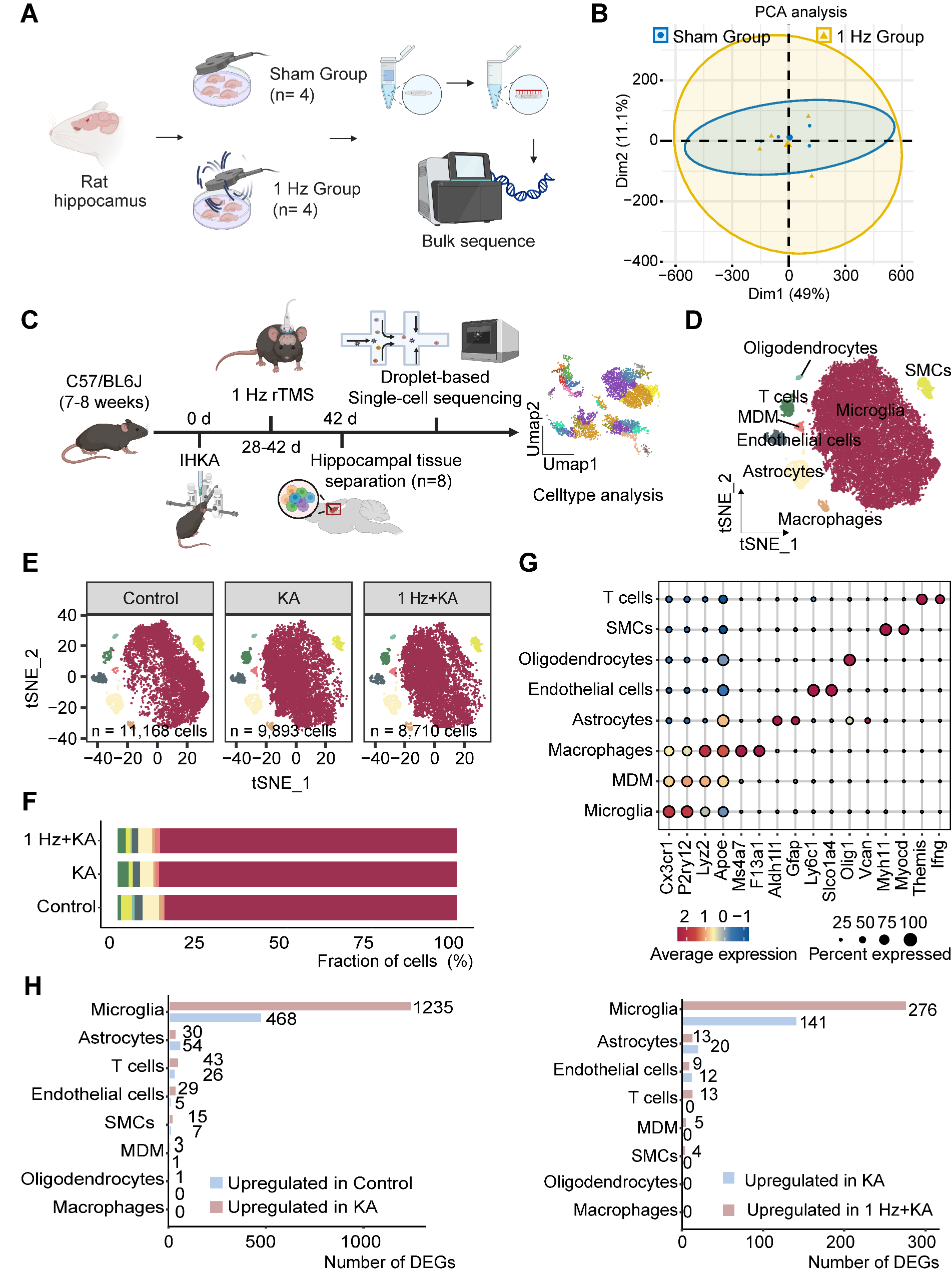


**Fig. S1** Integrated transcriptomic overview of bulk RNA-seq and single-cell RNA-seq datasets in the rodent hippocampus following 1 Hz LF-Rtms. **(A)** Schematic of bulk RNA sequencing for rat hippocampal tissue following in vitro 1 Hz LF-rTMS intervention. n = 4 rats per group. **(B)** PCA of bulk RNAseq shown there is no significant transcriptomic difference between the 1 Hz LF-rTMS intervention group and the sham control group. **(C)** Schematic of the workflow used to generate the atlas. n = 8 mice per group. **(D)** t-SNE of all cells in the atlas. **(E-F)** t-SNE showing cell distributions at control, KA, and 1 Hz group. And stacked barplot depicting the proportions of cells in the reference atlas from each group. **(G)** Bubble plot illustrates that cells are divided into eight cell types based on dominant gene expression. **(H)** DEGs counts per cell type (expressed in 25% of cells with |log FC| > 0.25). KA, kainic acid; MDM, monocyte-derived macrophages; SMCs, smooth muscle cells; DEGs, differential expressed genes.


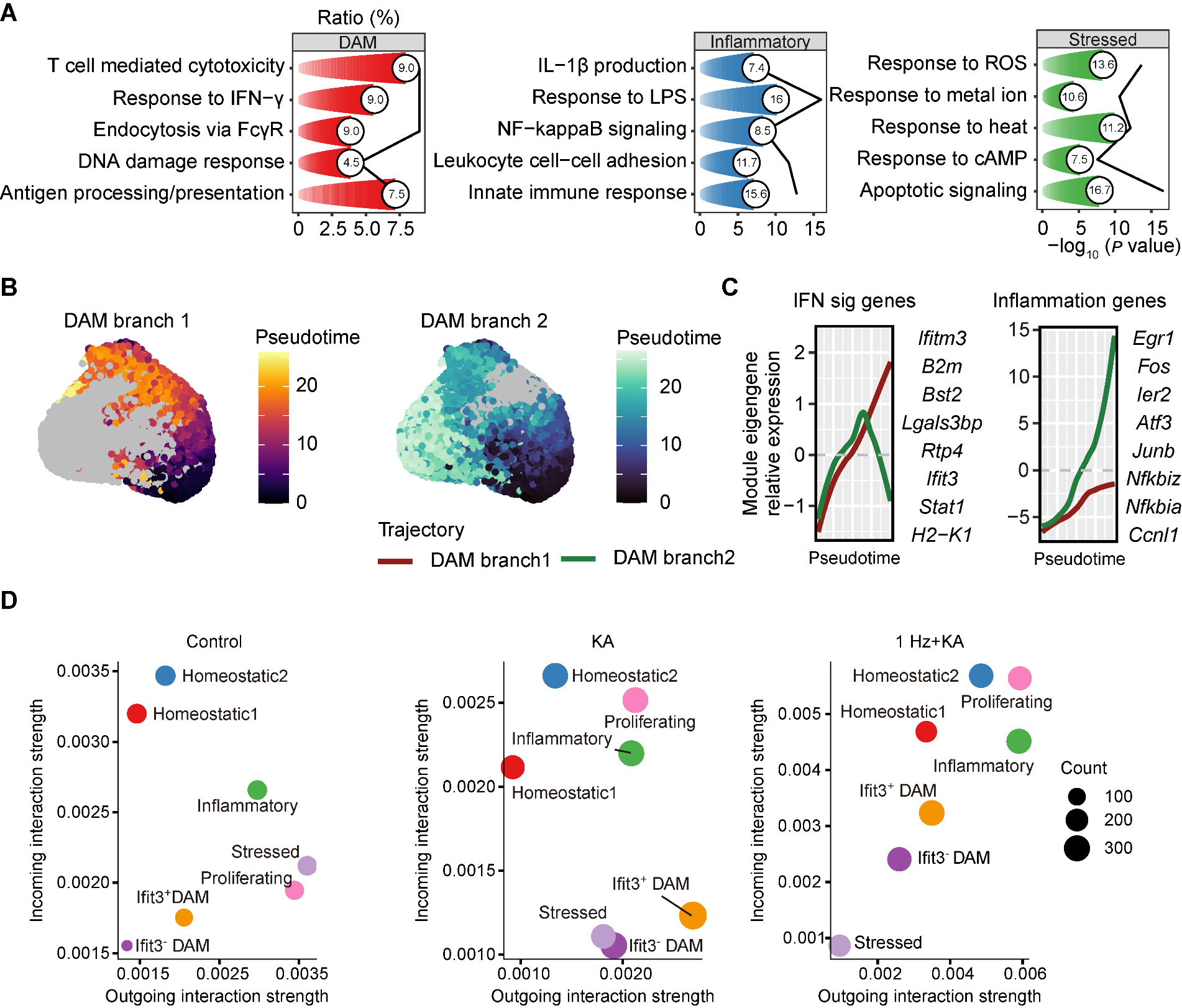


**Fig. S2** 1 Hz LF-rTMS isoform-dependent atlas of hippocampal microglia in epilepsy. **(A)** Enriched GO terms linked to the top 50 up-regulated DEGs in each subcluster of DAM, inflammatory, and stressed microglia. **(B)** Clustering of microglia and their two trajectories by Monocle3. **(C)** Expression level changes of IFN signature genes and inflammation genes associated with two DAM branch during Monocle3. **(D)** Scatter plots showing the incoming and outcoming interaction strength in each group. DAM, disease-associated microglia; FcγR, Fc-gamma Receptor; IFN sig genes, Interferon signature genes.


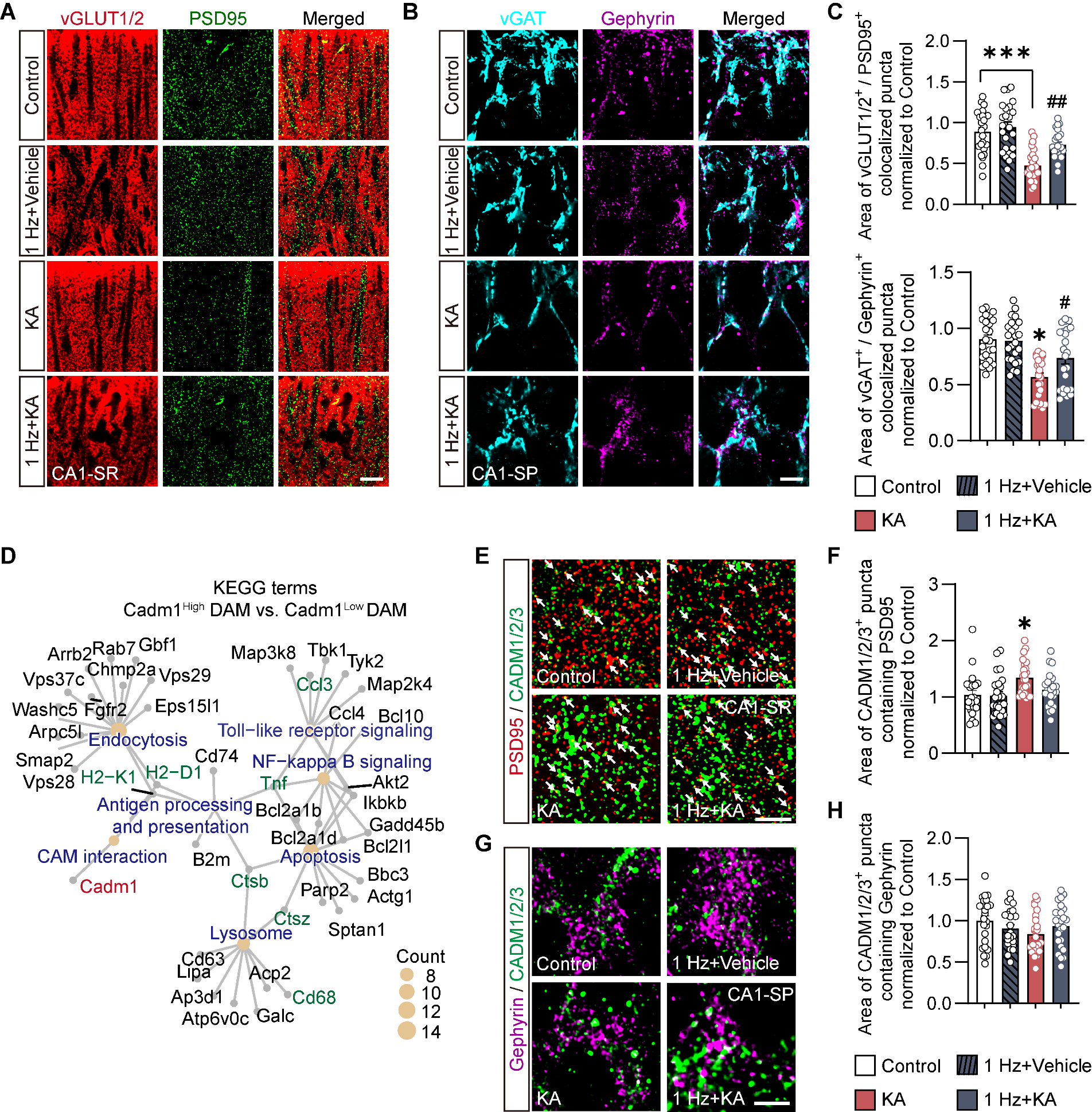


**Fig. S3** 1 Hz LF-rTMS treatment may regulate CADM1/2/3 expression to prevent the loss of excitatory synapses in the hippocampus of epileptic mice. **(A-B)** Immunofluorescence detection of inhibitory synapses and excitatory synapses in mouse hippocampus. Scale bar = 10 µm. **(A)** VGLUT1/2 (red) & PSD95 (green) double-staining. **(B)**vGAT (cyan) & Gephyrin (magenta) double-staining. **(C)** Quantification of vGLUT1/2&PSD95 positive excitatory synapses and vGAT/Gephyrin positive inhibitory synapses in CA1 are shown. n = 3 images from 8 mice/group. **(D)** Significant KEGGs and genes upregulated in high expression of Cadm1 group. Cadm1 with high expression (counts expression ≥2.0). Green nodes represent microglial genes associated with phagocytosis, neuroinflammation, and antigen presentation. **(E)** Immunofluorescence detection of CADM1/2/3 (green) contacting with PSD95^+^ excitatory post-synapses (red) in stratum radiatum. Scale bar = 5 µm. **(F)** quantification of the area of PSD95+ puncta contacted with CADM1/2/3 in each group. n = 3 images from 8 mice/group. **(G)** Immunofluorescence detection of CADM1/2/3 (green) contacting with Gephyrin^+^ excitatory post-synapses (magenta) in stratum pyramidale. Scale bar = 5 µm. **(H)** quantification of the area of Gephyrin^+^ puncta contacted with CADM1/2/3 in each group. n = 3 images from 8 mice/group. Data are shown as the means ± SEM. **p* < 0.05, ***p* < 0.01, ****p* < 0.001, compared to Control; ^##^*p* < 0.01, and ^###^*p* < 0.001. compared to KA; one-way ANOVA followed by Tukey’s post hoc tests. SR, stratum radiatum; SP, stratum pyramidale; KA, kainic acid; DAM, disease-associated microglia.


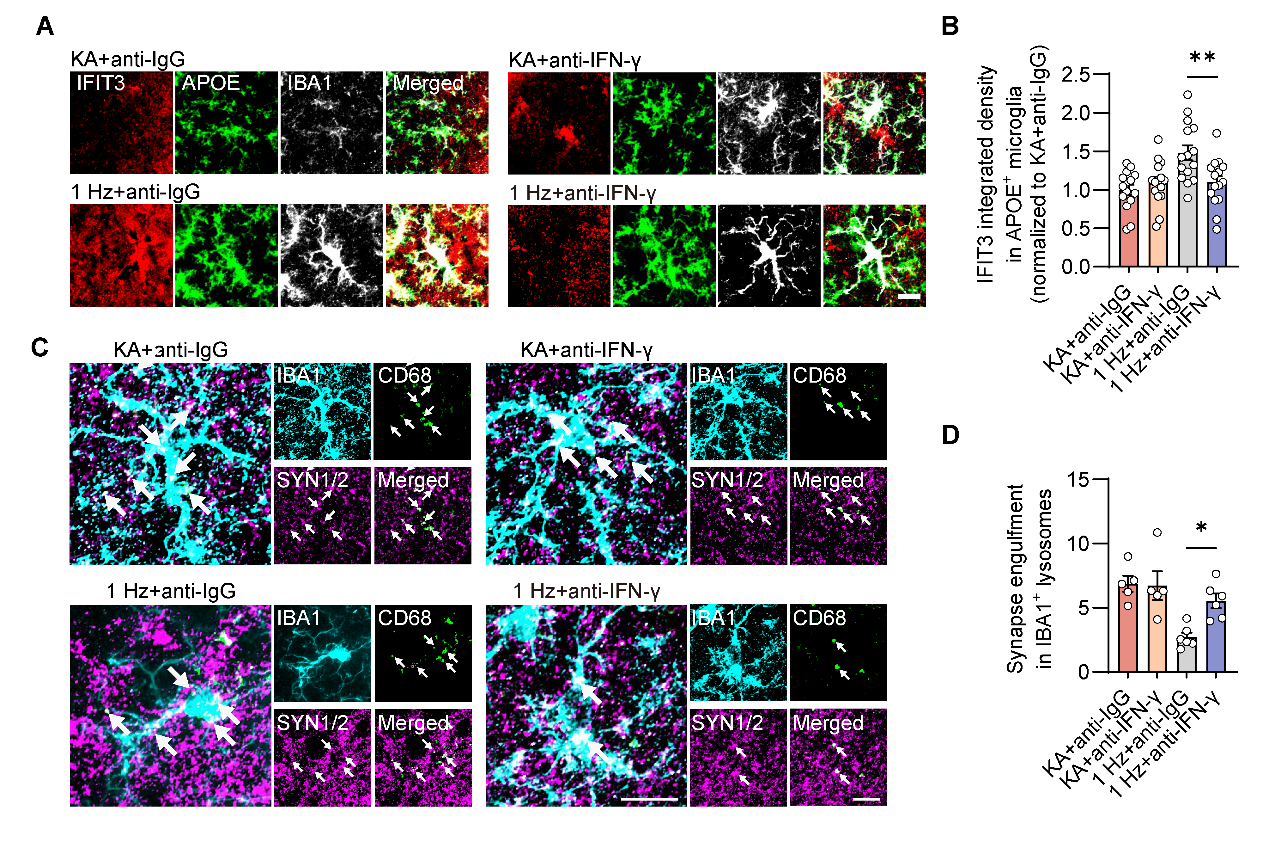


**Fig. S4** Inhibiting IFN-γ signaling negates the beneficial effects of 1 Hz LF-rTMS on microglial synaptic phagocytosis. **(A)** Triple immunostaining of IFIT3 (red), APOE (green), and IBA1 (white) indicates reduced IFIT3 expression in CA1 hippocampal microglia with IFN-γ blocking. **(B)** IFIT3 signal intensity in APOE^+^ microglia quantified. scale bar = 10 μm, n = 3 images from 5 mice/group. **(C)** Immunofluorescence shows synapses engulfed by microglia in CA1, with CD68 (blue), SYN1/2 (magenta), and IBA1 (cyan) labeling. scale bar = 10 μm. **(D)** Blocking IFN-γ signaling may reduce 1 Hz LF-rTMS's effect on SE-induced synaptic phagocytosis by microglia. n = 5-6 mice/group. Data are shown as the means ± SEM. **p* < 0.05, ***p* < 0.01, ****p* < 0.001, compared to 1 Hz+anti-IgG, one-way ANOVA followed by Tukey’s post hoc tests. KA, kainic acid; SYN1/2, synapsin1/2.
